# Differential effects of dietary protein sources on postprandial low-grade inflammation after a single high fat meal in obese non-diabetic subjects

**DOI:** 10.1186/1475-2891-10-115

**Published:** 2011-10-19

**Authors:** Jens Holmer-Jensen, Toni Karhu, Lene S Mortensen, Steen B Pedersen, Karl-Heinz Herzig, Kjeld Hermansen

**Affiliations:** 1 Department of Endocrinology and Metabolism MEA, Aarhus University Hospital, Aarhus, Denmark; 2 Institute of Biomedicine and Biocenter of Oulu Faculty of Medicine, Finland

**Keywords:** obesity, metabolic syndrome, dietary protein, nutrition, inflammation, cytokines, postprandial period

## Abstract

**Background:**

Obesity is a state of chronic low-grade inflammation. Chronic low-grade inflammation is associated with the pathophysiology of both type-2 diabetes and atherosclerosis. Prevention or reduction of chronic low-grade inflammation may be advantageous in relation to obesity related co-morbidity. In this study we investigated the acute effect of dietary protein sources on postprandial low-grade inflammatory markers after a high-fat meal in obese non-diabetic subjects.

**Methods:**

We conducted a randomized, acute clinical intervention study in a crossover design. We supplemented a fat rich mixed meal with one of four dietary proteins - cod protein, whey isolate, gluten or casein. 11 obese non-diabetic subjects (age: 40-68, BMI: 30.3-42.0 kg/m2) participated and blood samples were drawn in the 4 h postprandial period. Adiponectin was estimated by ELISA methods and cytokines were analyzed by multiplex assay.

**Results:**

MCP-1 and CCL5/RANTES displayed significant postprandial dynamics. CCL5/RANTES initially increased after all meals, but overall CCL5/RANTES incremental area under the curve (iAUC) was significantly lower after the whey meal compared with the cod and casein meals (*P *= 0.0053). MCP-1 was initially suppressed after all protein meals. However, the iAUC was significantly higher after whey meal compared to the cod and gluten meals (*P *= 0.04).

**Conclusion:**

We have demonstrated acute differential effects on postprandial low grade inflammation of four dietary proteins in obese non-diabetic subjects. CCL5/RANTES initially increased after all meals but the smallest overall postprandial increase was observed after the whey meal. MCP-1 was initially suppressed after all 4 protein meals and the whey meal caused the smallest overall postprandial suppression.

**Trial Registration:**

ClinicalTrials.gov ID: NCT00863564

## Background

The global incidence of obesity is escalating at epidemic proportions. The obesity related co-morbidities include increased incidence of the metabolic syndrome, type-2 diabetes (T2DM), hypertension, dyslipidaemia and chronic low-grade inflammation [1,2].

Interestingly, Hotamisligil *et al *[3] in 1993 suggested that chronic low-grade inflammation plays a role in the pathophysiology of obesity in general and of insulin resistance in particular. This has subsequently been supported by the demonstration of a correlation between circulating levels of inflammatory markers and both T2DM [4] and atherosclerosis in humans [5-8].

White adipose tissue (WAT) is an important endocrine organ that secretes molecules, referred to as adipokines [9]. The chronic low-grade inflammation of obesity is characterized by increased concentrations of circulating inflammatory adipokines and cytokines [10-13]. Importantly, the inflammatory and metabolic pathways are linked. WAT is infiltrated with macrophages in response to adipocyte hypertrophy and the associated increase in monocyte chemotactic protein-1 (MCP-1) expression [14,15]. Increased circulating concentrations of MCP-1 are in humans predictive of both diabetes risk independently of other traditional risk factors [16] and atherosclerosis [17,18]. Furthermore, differentiation of monocytes into macrophages starts in plasma, when monocytes are activated in response to postprandial triglyceride rich lipoproteins [19,20] and free fatty acids [21]. While MCP-1 is now regarded as a key inflammatory marker, CC chemokine ligand-5 (CCL5/RANTES) has in recent years emerged as a potentially therapeutic target in the prevention of atherosclerosis [22,23]. The interaction between CCL5/RANTES and monocytes facilitates the adherence and transmigration of monocytes through the arterial wall [22] initiating the atherosclerotic process. CCL5/RANTES antagonisms have been demonstrated to reduce atherosclerotic lesions in mice [24]. Furthermore, CCL5/RANTES is up-regulated in WAT of obese compared to lean subjects [25] facilitating macrophage infiltration of adipose tissue.

Though, the impact of dietary protein on postprandial inflammation has not been thoroughly elucidated, the impact of diet in general on low-grade inflammation has been demonstrated (reviewed in [26]). Thus, a positive correlation to postprandial inflammation has been demonstrated for total energy intake in healthy men [27,28] and a diet rich in saturated fat in overweight subjects [29]. Moreover dyslipidaemia characteristically for obesity, i.e. hypertriglyceridaemia, elevated apolipoprotein (Apo) B and small, dense low-density lipoproteins (LDL) has been positively correlated to low-grade inflammation in abdominally obese subjects with and without the metabolic syndrome [30]. The composition of meal fatty acids play an important role for low-grade inflammation in humans i.e. n-3 polyunsaturated fatty acids (PUFA) being anti-inflammatory while n-6 PUFA and saturated fatty acids appear to be pro-inflammatory [31-33]. The impact of dietary carbohydrate on postprandial inflammation is controversial [26,34].

Less is known about the influence of dietary protein on postprandial inflammation. Arya *et al *[35] demonstrated that meat with high fat content is more pro-inflammatory compared to lean meat in healthy subjects. Moreover, an inverse relationship has been demonstrated between dairy product consumption and low-grade inflammation in healthy subjects [36] and obese subjects [37].

We hypothesize that dietary protein sources may exert a differential impact on acute postprandial low-grade inflammation. In the present study we focused on the two inflammatory markers MCP-1 and CCL5/RANTES. As cod protein, gluten, casein and whey protein have been demonstrated to elicit differential postprandial lipid, glucose and hormone responses in healthy [38], overweight [39] and type-2 diabetic subjects [40] these four protein sources originating from fish, crops and milk may be suitable for assessing the impact of dietary protein on postprandial low-grade inflammation. The differential impact of the four protein sources on postprandial triglycerides and insulin may particularly reveal correlated differential responses on postprandial low-grade inflammation.

## Subjects and methods

11 obese Caucasian subjects (8 postmenopausal women and 3 men) were recruited after advertising in local newspapers. All subjects had a body mass index (BMI) above 30 and all subjects were non-diabetics according to fasting plasma glucose < 7.0 mmol/l. Subjects with impaired fasting glucose were subjected to an oral glucose tolerance test and were excluded if the 2 h plasma glucose level was ≥ 11.1 mmol/l. No participant took medication with impact on lipids, inflammation, immune system or insulin sensitivity and all participants were non-smokers. No change in concomitant medication was allowed during the trial. Subject characteristics are shown in Table 1. All subjects gave written informed consent and the study was approved by The Committees on Biomedical Research Ethics for the Central Region of Denmark. This study was registered at clinicaltrials.gov (ID: NCT00863564).

**Table 1:** Clinical characteristics of the eleven (8 women and 3 men) obese non-diabetic subjects^1^

Age (yr)	55.2 ± 9.4 (40-68)
Weight (kg)	100.9 ± 13.8 (79.0-120.9)
BMI (kg/m^2^)	33.9 ± 3.4 (30.3-42.0)
Waist (cm)	111.4 ± 6.8 (102-121)
♂	118.7 ± 2.1 (117-121)
♀	108.6 ± 5.7 (102-117)
Waist-to-hip ratio	0.92 ± 0.07 (0.83-1.08)
♂	1.01 ± 0.06 (0.98-1.08)
♀	0.89 ± 0.03 (0.83-0.92)
HbA1c (%)	5.8 ± 0.4 (5.3-6.5)
HOMA2 (IR)	1.3 ± 0.5 (0.3-2.0)
Fasting plasma glucose (mmol/l)	5.9 ± 0.4 (5.3-6.6)
Fasting plasma triglyceride (mmol/l)	2.0 ± 0.8 (0.7-3.1)

^*1 *^All values are means ± SD; range in parentheses.

### Study design

We performed a nested randomized, acute clinical intervention study. All subjects ingested four different meals on four different days with a washout period of two weeks between meals. Each subject was randomized to one of four meal sequences based on a Latin square design. Before each test day the subjects were given and consumed a standard diet with the following energy distribution: 56% energy from carbohydrate, 24% energy from fat and 20% energy from protein. The energy content was 7 000 kJ for women and 9 000 kJ for men. All subjects were asked to refrain from alcohol consumption and exercise in the 24 h preceding the test day. In the morning after a 12 h fasting period a catheter was inserted into an antecubital vein. After 15 min of rest baseline samples were drawn. The test meal was served and ingested within 20 min and blood samples for inflammatory markers were drawn in the 4 h postprandial period. The subjects were allowed to drink tap water *ad libitum*. Plasma was separated immediately by centrifugation at 2 000 × g for 20 min at 4°C. Plasma samples were stored at -80°C until analyzed.

### Test meals

All subjects consumed in random order four fat rich test meals containing 4971 - 4986 KJ with 19 E% carbohydrate, 66 E% fat and 15 E% protein, respectively. All test meals consisted of an energy free soup with an added 100 g of butter (Lurpak; Arla Foods amba, Viby J, Denmark) corresponding to 80 g of fat (68% of energy as saturated fat). 45 g of carbohydrate was added as white wheat bread (Läntmann Schulstad A/S, Hvidovre, Denmark) and 45 g of a protein preparation was added or served with the meal. The protein sources were cod, whey isolate, gluten and casein. The 45 g of cod protein (cod meal) corresponded to 285 g of minced cod filet (Coop torskefilet; Royal Greenland A/S, Aalborg, Denmark). This was added to the soup before heating. The spray dried whey isolate (whey meal) (Lacprodan DI-9224; kindly provided by Arla Foods Ingredients amb, Viby J, Denmark) was dissolved in 200 ml water and served with the meal. Gluten (gluten meal) (Gluvital 21000; kindly provided by Cerestar Scandinavia A/S, Charlottenlund, Denmark) was mixed into the soup before heating. Casein (casein meal) was applied as spray dried calcium caseinate (Miprodan 40; kindly provided by Arla Foods Ingredients amba, Viby J, Denmark). Half of the casein was dissolved in water and the other half was added to the soup before heating. To make the soup more palatable we added 25 g of sliced raw leek, 1 g of curry and ½ dice of chicken bouillon. The soup had a serving temperature of 65°C. The total amount of water in each meal was 675 ml and the total volume of each meal did not differ.

### Blood analyses

Adiponectin was measured using the B-Bridge Int. human adiponectin enzyme-linked immunosorbant assay (ELISA) kit (Cat# K1001-1), (CV: 3.2%). All other inflammatory markers were assessed using a fixed Bio-Plex Pro Human Cytokine 27-plex array (Cat# M50-0KCAF0Y) according to manufacturer's instructions, (CV: 5-15%) as described previously [33,41]. Plasma samples were diluted 1:2 and incubated with anti-cytokine antibody-coupled beads for 30 min at room temperature. Beads were then incubated with biotinylated detection antibodies for 30 min, before incubation with Streptavidin-phycoerythrin for 30 min. Following each incubation step, complexes were washed 3 times in wash buffer using the Bio-Plex Pro Wash Station. Finally, complexes were resuspended in 125 μl of assay buffer, and beads were counted on the Bio-Plex 200 system (Bio-Rad). Duplicates were performed. Mean fluorescence intensity was analyzed and data was given as pg/ml. The 27-plex array included the following cytokines: Interleukine (IL)-1b, IL-1 receptor antagonist (IL-1ra), IL-2, IL-4, IL-5, IL-6, IL-7, IL-8, IL-9, IL-10, IL-12 (p70), IL-13, IL-15, IL-17A, basic fibroblast growth factor (basic FGF), eotaxin, granulocyte colony stimulating factor (G-CSF), granulocyte macrophage colony stimulating factor (GM-CSF), interferon-γ (IFN-γ), IFN-γ induced protein 10 kDa (IP-10), MCP-1, macrophage inflammatory protein-1α (MIP-1α), MIP-1β, platelet derived growth factor BB (PDGF-BB), CCL5/RANTES, Tumor necrosis factor-α (TNF-α), vascular endothelial growth factor (VEGF).

### Statistical analysis and calculations

Comparisons were based on a mixed effects model [42] (STATA/IC 10.1) using treatment group as fixed variable and participant ID as random variable. All estimates were adjusted for treatment order, baseline values, gender and waist-to-hip ratio. F-test or Wald test were applied as appropriate. A *P *value < 0.05 was considered statistically significant. Any statistical significant main effect of meal was followed up by Tukey's post hoc correction for pair wise comparison. Whenever data was not normally distributed, a log transformation was performed and the statistical analysis was carried out on the normal distributed log data. Data was given as net incremental area under the curve (iAUC) 0-240 min. Net iAUC was calculated using trapezoidal rule. Data was given as means ± SD in tables and as means ± SE in graphs unless otherwise stated. When statistical analyses were performed on log transformed data, results were given as medians with interquartile ranges. Any sample with a value below detection limit was omitted from the statistical analyses. No statistical analyses were performed on cytokines with more than 9% of samples below detection limit: IL-1b, IL-2, IL-4, IL-5, IL-6, IL-7, IL-8, IL-9, IL-10, IL-12 (p70), IL-13, IL-15, IL-17A, basic FGF, GM-CSF, MIP-1α and TNF-α.

## Results

The 11 obese non-diabetic subjects completed the four test meals according to protocol. No significant differences in fasting cytokines concentrations were found (Table 2). No significant weight changes were found between first (100.5 kg) and last (100.4 kg) test day.

**Table 2:** Fasting concentrations and net incremental areas under the curve after 240 min (net iAUC 0-240 min)^1 ^for inflammatory markers, insulin and triglycerides in 11 (8 women and 3 men) obese non-diabetic subjects in response to the four test-meals

	Cod	Whey	Gluten	Casein
MCP-1				
Fasting	22 ± 11	21 ± 7	23 ± 12	21 ± 11
iAUC 0-240 min (pg/ml·240 min)	- 826 ± 1255x	- 77 ± 1092y	-943 ± 925x	-469 ± 757xy
CCL5				
Fasting	340 ± 238	490 ± 396	490 ± 321	409 ± 297
iAUC 0-240 min (pg/ml·240 min)	50 764 ± 58 218x	- 18 510 ± 81 999y	11 235 ± 55 558yz	44 488 ± 37 862xz
IL-1ra				
Fasting	68 ± 18	79 ± 39	76 ± 34	66 ± 24
iAUC 0-240 min (pg/ml·240 min)	743 ± 2 871	-1 972 ± 4 005	-1 454 ± 5 068	2 173 ± 4 320
PDGF-bb1				
Fasting	40 ± 41	161 ± 260	65 ± 78	110 ± 232
iAUC 0-240 min (pg/ml·240 min)	11 837 (6 285 - 20 952)	4 303 (2 102 - 6 995)	6 660 (4851 - 10 742)	8 604 (6348 - 10 435)
IFN-g				
Fasting	25 ± 12	28 ± 25	30 ± 21	28 ± 24
iAUC 0-240 min (pg/ml·240 min)	865 ± 1 610	-1 300 ± 3 747	-791 ± 2 622	397 ± 2 614
Adiponectin				
Fasting	1.5 ± 0.5	1.5 ± 0.6	1.5 ± 0.6	1.5 ± 0.5
iAUC 0-240 min (μg/ml·240 min)	-7.7 ± 48	-8.5 ± 49	6.3 ± 38	-6.9 ± 36
Eotaxin				
Fasting	52 ± 12	53 ± 14	51 ± 14	49 ± 16
iAUC 0-240 min (pg/ml·240 min)	- 45 ± 819	601 ± 2237	- 64 ± 686	983 ± 1039
G-CSF				
Fasting	11 ± 3	12 ± 6	11 ± 4	12 ± 7
iAUC 0-240 min (pg/ml·240 min)	23 ± 719	-420 ± 1 242	-114 ± 685	-194 ± 955
IP-10				
Fasting	579 ± 166	558 ± 109	588 ± 226	542 ± 142
iAUC 0-240 min (pg/ml·240 min)	-2 343 ± 24 198	-10 371 ± 19 737	-3 403 ± 21 448	-5 159 ± 10 121
MIP-1b				
Fasting	45 ± 16	46 ± 16	44 ± 14	43 ± 15
iAUC 0-240 min (pg/ml·240 min)	-908 ± 1 248	-331 ± 1 411	-302 ± 534	-219 ± 428
VEGF				
Fasting	7 ± 3	10 ± 6	7 ± 3	8 ± 4
iAUC 0-240 min (pg/ml·240 min)	693 ± 921	270 ± 1 058	233 ± 448	317 ± 419
Insulin				
Fasting	65 ± 28	63 ± 33	66 ± 31	62 ± 32
iAUC 0-240 min (pmol/l·240 min)	41 158 ± 11 003x	73 693 ± 28 022y	38 738 ± 10 691x	50 978 ± 21 477x
Triglycerides				
Fasting	2.0 ± 1.0	1.8 ± 0.8	1.9 ± 0.7	1.8 ± 0.9
iAUC 0-240 min (mmol/l·240 min)	138 ± 108	122 ± 95	132 ± 95	127 ± 94

^1^All values are means ± SDs (normal distributions; mixed-effects model, Tukey's *post hoc *correction) or medians with interquartile ranges in parentheses (skewed distribution; mixed-effects model on normal distributed log-transformed data, Tukey's *post hoc *correction). Meals: energy-free soup plus 100 g butter and 45 g carbohydrate consumed with either 45 g cod, 45 g whey isolate, 45 g gluten or 45 g casein. MCP-1: monocyte chemotactic protein-1; CCL5: Chemokine ligand 5; IL-1ra: interleukin 1 receptor-antagonist; PDGF-bb1: platelet derived growth factor BB; IFN-g: interferon-gamma; G-CSF: granulocyte colony-stimulating factor; IP-10: interferon-gamma induced protein 10 kDa; MIP-1b: macrophage inflammatory protein 1b; VEGF: vascular endothelial growth factor. Values in a row with different superscript letters are significantly different, *P *< 0.05.

### CCL5/RANTES

**Figure 1:**
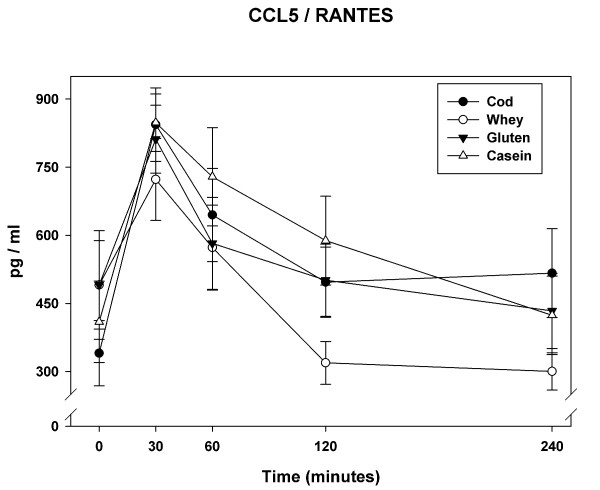
**The plot show mean (+SEM) responses for CCL5/RANTES in plasma in the 4 h postprandial period after the four meals consumed by 11 obese non-diabetic subjects**. Meals consisted of an energy-free soup plus 80 g fat (from butter) and 45 g carbohydrate consumed with either 45 g cod protein, 45 g whey protein, 45 g gluten or 45 g casein.

The plasma concentrations of CCL5/RANTES increased for all meals at 30 min (Figure 1). The average increment at 30 min was 86%. Towards the end of the observation period CCL5/RANTES concentrations decreased towards baseline for all meals. At 240 min CCL5/RANTES concentration after cod-meal was 52% above baseline whereas CCL5/RANTES concentration after whey-meal was 39% below baseline. We found a statistically significant main effect of meal (*P *= 0.0053). The iAUC-240 min was lower after whey-meal compared to cod meal and casein meal. The iAUC-240 min was also lower after gluten meal compared to cod meal (Table 2). *Post hoc *analyses did not demonstrate differences between meals in CCL5/RANTES iAUC-60 min (*P *= 0.55). However, a negative correlation was demonstrated between insulin iAUC-240 min and CCL5/RANTES iAUC-240 min (*r = *-0.33; *P = *0.04). No *post hoc *correlation could be demonstrated between triglycerides and MCP-1 (data not presented).

### MCP-1

**Figure 2:**
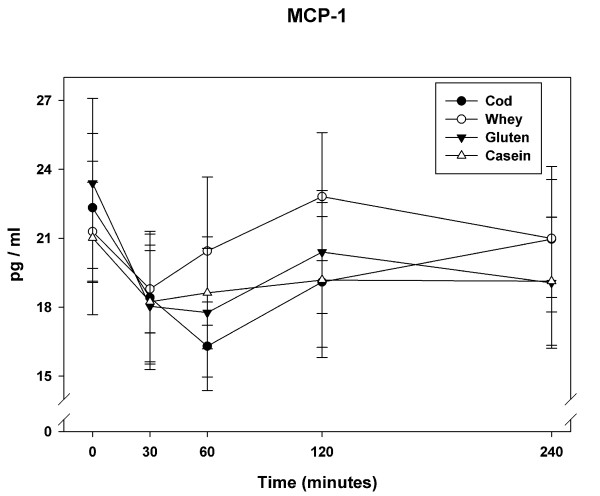
**The plot show mean (+SEM) responses for MCP-1 in plasma in the 4 h postprandial period after the four meals consumed by 11 obese non-diabetic subjects**. Meals consisted of an energy-free soup plus 80 g fat (from butter) and 45 g carbohydrate consumed with either 45 g cod protein, 45 g whey protein, 45 g gluten or 45 g casein.

MCP-1 plasma levels decreased for all meals in the first 30 min (Figure 2). The mean decrement at 30 min was 17%. Towards the end of the observation period MCP-1 levels after whey-meal reached baseline whereas MCP-1 concentrations only slightly increased but remained below baseline for the three other meals. We found a statistically significant main effect of meal (*P *= 0.040). The overall net suppression of MCP-1 was smaller for whey-meal compared to cod-meal and gluten-meal (Table 2). *Post hoc *analyses did not demonstrate differences between meals in MCP-1 iAUC-60 min (*P *= 0.38). However, a *post hoc *correlation analysis revealed a positive correlation between insulin iAUC-240 min and MCP-1 iAUC-240 min (*r = *0.39; *P = *0.01). No *post hoc *correlation could be demonstrated between triglycerides and MCP-1 (data not presented).

### Other cytokines

No significant differences between meals were observed for IFN-γ, adiponectin, eotaxin, G-CSF, IP-10, MIP-1β, PDGF-BB, IL-1ra and VEGF, respectively (Table 2).

## Discussion

We have demonstrated acute differential effects of dietary protein sources on postprandial low-grade inflammation after a high-fat meal in obese non-diabetic subjects. Of particular interest we observed that MCP-1 and CCL5/RANTES displayed acute postprandial responses to the test meals. MCP-1 was initially suppressed and CCL5/RANTES initially increased after consumption of the test meals. For both cytokines no significant differences between meals were evident at peak values after 60 min. However, whey-meal was associated with a different overall response after 240 min compared to the other protein meals. MCP-1 was suppressed to a smaller extent after whey-meal compared to cod-meal and gluten-meal. CCL5/RANTES iAUC-240 min was smaller after the whey-meal compared to the other meals - in particular compared to cod-meal and casein-meal.

Several studies have demonstrated postprandial adherence of Apo B to monocytes and activation of monocytes in response to an oral fat loading test in healthy subjects [19,20]. The activation of monocytes is important for the endothelial adhesion of monocytes and subsequent transmigration across the endothelial wall where the monocytes differentiate into macrophages [43]. However, this process is further enhanced when oxidized LDL and chylomicron remnant particles are taken up by residing macrophages inside the vessel wall. These macrophages activate the endothelium to produce MCP-1 which in mice resulted in further localized recruitment and tissue infiltration of monocytes [44]. In the endothelial wall the phagocytosis of oxidized lipoproteins by macrophages precedes the development of atherosclerotic plaques. Consequently any means to reduce endothelial adhesion of monocytes may reduce the progression of atherosclerotic plaques.

The CCL5/RANTES response was significantly smaller after whey meal compared to the cod and casein meals. Krohn *et al *[45] demonstrated reduction of neointimal thickening and macrophage infiltration in CCL5/RANTES knock-out mice. These findings are consistent with the CCL5/RANTES antagonist study of Braunersreuther *et al *[46] who also demonstrated that deficiency of the CCL5/RANTES receptor protects Apo E -/- mice from diet induced atherosclerosis [47]. The finding of up-regulated CCL5/RANTES in human atherosclerotic plaques [48] corroborates with the association demonstrated between CCL5/RANTES and unstable angina pectoris [49] and myocardial infarction [50]. While CCL5/RANTES is thought to be crucial to monocyte recruitment during development of atherosclerosis [51] high density lipoprotein may partly cause its cardioprotective effect by reducing circulating levels of CCL5/RANTES [52]. Furthermore, high levels of CCL5/RANTES had a positive correlation to the development of T2DM in humans [53].

In the present study cod protein and gluten induced significantly lower concentrations of MCP-1 compared to whey protein. The mechanism of action is not known. However, in the cod-meal the total quantity of n-3 fatty acids was 752 mg, which may contribute to the anti-inflammatory effects via interaction with the pro-inflammatory transcription factor i.e. nuclear factor kappa beta (NF-κB) [54,55]. However, since gluten does not contain any n-3 fatty acids it may be speculated that cod and gluten do not share particular MCP-1 lowering properties. MCP-1 may be higher after whey meal due to whey specific properties e.g. higher insulin response as discussed later.

Research on the immunomodulatory properties of milk proteins has traditionally been focusing on the antimicrobial effects of T-cells, macrophages and the innate immune response [56]. The availability of immunomodulatory peptides is not solely depending on the dietary composition but also varies depending on the specific enzymatic digestion of milk components in the intestinal tractus [57]. Aihara *et al *[58] demonstrated that the casein and whey derived tri-peptide valyl-prolyl-proline modulates monocyte adhesion to inflamed endothelia *in vitro *via attenuation of the pro-inflammatory c-Jun N-terminal kinases (JNK) pathway. Interestingly, the casein subunit "α s1" is expressed by monocytes *in vitro *[59] promoting a pro-inflammatory cytokine response. Furthermore, Zemel *et al *[36] demonstrated reduced levels of MCP-1 after a dairy rich diet but not after a soy rich diet in a 28 day intervention period. These observations support that circulating peptides from a dairy product rich diet may at least in part be responsible for the differential cytokine responses observed after the four meals in the present study.

Whey protein reduces postprandial lipaemia more than cod, gluten and casein [39,40]. However, we could not demonstrate any correlation between postprandial lipaemia and the inflammatory markers.

Euglycemic hyperinsulineamia has been found to inhibit NF-κB and reduce concentrations of MCP-1 in obese subjects after 2 h and 4 h of insulin infusion [60,61]. As whey protein is more insulinotropic than cod protein, gluten and casein this mechanism would imply a larger suppression of MCP-1 after the whey meal compared to the other meals. However, we have demonstrated a positive correlation between postprandial insulin and MCP-1 as well as a negative correlation between postprandial insulin and CCL5/RANTES. This is in accordance with our findings that the MCP-1 iAUC-240 min for the whey meal was larger compared to the other meals and that the CCL5/RANTES iAUC-240 min was smaller after whey meal. Other studies have demonstrated anti-inflammatory properties of insulin infusion on both MCP-1 and CCL5/RANTES [62]. We cannot explain the opposing effects of whey meal on MCP-1 and CCL5/RANTES in our study.

## Conclusion

MCP-1 and CCL5/RANTES are risk markers closely associated to obesity related risk factors, i.e. dyslipidaemia and insulin resistance. Long-term studies are needed to establish the potentially clinical effect of the impact of dietary protein on postprandial low-grade inflammation. In the present study we demonstrated an inverse relationship between concentrations of postprandial MCP-1 and CCL5/RANTES after the cod, whey isolate, gluten and casein meals.

This study is an exploratory pilot study indicating differential effects of dietary protein sources on postprandial inflammatory cytokines. Inclusion of different protein rich foods may enhance or diminish the inflammatory properties of a given diet. As circulating concentrations of MCP-1 and CCL5/RANTES are profoundly affected in the postprandial period, future research on postprandial low-grade inflammation should include these key inflammatory markers.

## List of abbreviations

Apo: apo protein; BMI: body mass index; CCL5: CC chemokine ligand-5; ELISA: enzyme-linked immunosorbent assay; FGF: fibroblast growth factor; G-CSF: granulocyte colony stimulating factor; GM-CSF: granulocyte macrophage colony stimulating factor; iAUC: incremental area under the curve; IFN-γ: interferon-γ; IL: interleukine; IL-1ra: IL-1 receptor antagonist; IP-10: interferon-γ induced protein 10 kDa; JNK: c-Jun N-terminal kinases; LDL: low density lipoproteins; MCP-1: monocyte chemotactic protein-1; MIP-1α: macrophage inflammatory protein-1α; NF-κB: nuclear factor kappa beta; PDGF-BB: platelet derived growth factor BB; PUFA: poly unsaturated fatty acids; RANTES: regulated upon activation; normal T-cell expressed; and secreted; T2DM: type 2 diabetes; TNF-α: tumor necrosis factor-α; VEGF: vascular endothelial growth factor; WAT: white adipose tissue.

## Conflict of interests

The authors declare that they have no competing interests.

## Authors' contributions

JHJ, TK, KHH and KH designed the study. JHJ and LSM recruited subjects and collected the data. SBP, TK and KHH performed the blood analyses. JHJ performed the data analysis. JHJ was responsible for drafting the manuscript; KH assisted with the editing of the manuscript. JHJ had primary responsibility for final content. All authors participated in the discussion of the results and commented on the manuscript. All authors read and approved the final manuscript.
